# Drug Clearance and Dosing During CytoSorb Hemoadsorption: A Systematic Review

**DOI:** 10.1097/CCE.0000000000001444

**Published:** 2026-07-14

**Authors:** Teresa Klaus, Patrick M. Honoré, Gabriella Bottari, Silvia De Rosa, Detlef Kindgen-Milles, Antoine Schneider, Nandor Marczin, Harriet Adamson, Joerg Scheier, Efthymios N. Deliargyris, Thomas D. Nolin

**Affiliations:** 1 Medical Affairs Department, CytoSorbents Europe GmbH, Berlin, Germany.; 2 Intensive Care Unit Department, CHUUCL Namur Mont-Godinne, UCL Louvain Medical School, Yvoir, Belgium.; 3 Pediatric Intensive Care Unit, Children Hospital Bambino Gesù, IRCCS, Rome, Italy.; 4 Anesthesia and Intensive Care Department, Centre for Medical Sciences (CISMed), University of Trento, Trento, Italy.; 5 Department for Anesthesiology, Medical Faculty, Heinrich Heine University Hospital, Düsseldorf, Germany.; 6 Adult Intensive Care Department, Centre Hospitalier Universitaire Vaudois (CHUV), Lausanne, Switzerland.; 7 Division of Anaesthesia, Pain Medicine and Intensive Care, Imperial College London, London, United Kingdom.; 8 Medical Affairs Department, CytoSorbents Corporation and CytoSorbents Medical, Princeton, NJ.; 9 Department of Pharmacy and Therapeutics, Department of Medicine Renal-Electrolyte Division, University of Pittsburgh, Pittsburgh, PA.

**Keywords:** antithrombotics, CytoSorb, extracorporeal clearance, hemoadsorption, hemoperfusion, pharmacodynamic, pharmacokinetic, therapeutic drug monitoring

## Abstract

**OBJECTIVE:**

CytoSorb (CS) hemoadsorption is increasingly used in critical care and cardiac surgery to remove inflammatory mediators and select drugs. This systematic review aimed to summarize available evidence on intentional drug removal as well as unintentional drug removal during CS therapy.

**DATA SOURCES:**

PubMed, congress abstracts, and CytoSorbents online literature database.

**STUDY SELECTION:**

Eligible studies included benchtop, animal, and human pharmacokinetic investigations of drug removal during CS therapy as well as clinical reports describing CS use for drug overdose.

**DATA EXTRACTION:**

Data from the eligible studies were extracted independently by two authors into Microsoft Excel.

**DATA SYNTHESIS:**

Data were identified for drugs across multiple therapeutic classes, including analgesics, antiarrhythmics, anticonvulsants, antidepressants, antihypertensives, anti-infectives, antithrombotics, anxiolytics, and immunosuppressants. Drugs were categorized into low (< 30%), moderate (30–60%), or high (> 60%) removal rate, or by the relative increase in clearance attributable to CS: negligible (< 25%), low (25–100%), moderate (100–400%), or high (> 400%). Based on the assembled data and considering drug-, patient-, and circuit-specific factors, general dosing recommendations for concomitant medications were developed.

**CONCLUSIONS:**

CS therapy can alter drug concentrations for select agents with variable clinical relevance. CS can reduce perioperative bleeding risk through antithrombotic removal and treat certain drug overdoses. Unintentional drug removal during CS therapy may occur and practical guidance is provided for drugs with known susceptibility to removal.

**KEY POINTS**

**Question:** What evidence exists regarding intentional and unintentional drug removal during CytoSorb therapy, and what practical dosing guidance can support safe concomitant drug administration?

**Findings:** This systematic review identified evidence for 72 drugs across multiple therapeutic classes. Most drugs showed minimal or no clinically relevant removal, whereas selected anti-infectives, antithrombotics, anticonvulsants, and psychotropic agents demonstrated potentially significant clearance. Drug removal is influenced by drug-related, patient-specific, and extracorporeal circuit factors. Practical guidance including dose timing, supplemental dosing, and therapeutic drug monitoring of concomitant medication has been developed.

**Meaning:** CytoSorb may significantly alter exposure for select drugs, requiring individualized dosing and monitoring strategies during therapy.

CytoSorb (CS, CytoSorbents, Princeton, NJ) is a hemoadsorption device used in critical care and cardiac surgery to remove select endogenous and exogenous substances from the blood. It is increasingly applied across a wide range of critical care scenarios, including sepsis (1), liver failure (2), rhabdomyolysis (3), severe burns (4), or during intra- and postoperative management in cardiac surgery (5). CS is a Conformité Européenne marked for the removal of cytokines, myoglobin, bilirubin, as well as for ticagrelor and rivaroxaban removal during cardiac surgery (6). Initial application of CS therapy was primarily alongside continuous renal replacement therapy (CRRT); however, over the years the feasibility for integration into multiple extracorporeal platforms was established thereby expanding CS use with extracorporeal life support, extracorporeal membrane oxygenation, and cardiopulmonary bypass (CPB) (7, 8).

Drug removal is a key consideration in patients receiving extracorporeal therapies as pharmacokinetic (PK) and pharmacodynamic (PD) profiles often change in complex clinical states (9). Organ dysfunction, fluid shifts, altered protein binding, and extracorporeal drug clearance contribute to variable drug exposure (10). Unfortunately, high-quality evidence on the impact of extracorporeal platforms on drug exposure remains limited.

There is a growing body of evidence supporting the use of CS for deliberate drug removal in specific clinical conditions (11, 12). Examples include intraoperative CS integration into CPB circuits for removal of ticagrelor or rivaroxaban during urgent cardiac surgery to reduce perioperative bleeding, as well as off-label CS use in cases of drug overdose and intoxications. In the latter scenario, often involving life-threatening concentrations of drugs with low dialyzability and without a specific antidote, CS has emerged as a potential detoxification strategy. Accordingly, such use has been reported in the management of overdoses of antidepressants, antiepileptics, and cardiovascular agents (11).

The potential for unintentional drug removal during extracorporeal hemoadsorption therapy must also be considered. This topic was addressed in a comprehensive review published in 2022 (13), but since then additional clinical and experimental data have emerged that offer new insights on the potential impact of unintended drug removal.

In the present review, we focus on the clinical implications for concomitant drug management, including intentional drug removal in select intoxications or perioperative settings and unintentional removal as a concomitant consideration during CS therapy. Because the clinical relevance of drug removal depends on the specific drug, patient condition, and extracorporeal context, practical guidance must remain individualized and supported by therapeutic drug monitoring whenever available.

## METHODS

The systematic review was registered in PROSPERO (CRD420251047956) and was conducted and reported in accordance with the Preferred Reporting Items for Systematic Reviews and Meta-Analyses (PRISMA) statement (14). A detailed search using the National Library of Medicine PubMed library (https://pubmed.ncbi.nlm.nih.gov/), congress abstracts and the device manufacturer’s online literature database (https://cytosorbents.com/lit-db/) was conducted on November 19, 2025. The CS literature database is publicly accessible and includes peer reviewed as well as non-peer reviewed publications (e.g., conference abstracts and posters) which, for the purpose of this review, were all included in the final analysis, if applicable. Search terms included “CytoSorb” and “drug removal.” Eligibility of studies for inclusion was cross-checked by two senior authors and full texts of eligible articles were then screened. Human, animal and bench-top studies with PK data during device therapy as well as publications that reported on CS treatments for drug overdose were selected for inclusion. Relevant PK data were examined and synthesized for narrative review. In-vivo results were prioritized over in vitro results.

Based on available data, drugs were categorized into low (< 30%), moderate (30–60%), or high rates of removal (> 60%), or, alternatively, according to acceleration of clearance relative to endogenous clearance into negligible (< 25%), low (25–100%), moderate (100–400%), or high (> 400%) (13, 15). In addition, if patients were treated concomitantly with other extracorporeal platforms, this was documented accordingly.

## RESULTS

In total, 167 articles were identified through PubMed (*n* = 49) and the company literature database (*n* = 118) searches. After duplicates and non-English articles were removed, 133 records were screened for eligibility. From these, 68 articles were excluded because they: a) did not contain original data (*n* = 29), b) did not report drug concentration levels (*n* = 32), or c) did not report details on device use (*n* = 7). For final analysis 65 publications were included (**Supplemental Fig. 1, PRISMA chart**, https://links.lww.com/CCX/B656) (14) including 23 from PubMed and 42 from the CS database. Of those identified in the CS database, 35 were peer reviewed articles and 7 congress abstracts, from which 5 also appear in journal supplements. Several publications reported on more than one drug, resulting in a total of 72 drugs included in this analysis, including analgesics, antiarrhythmics, anticonvulsants, antidepressants, antihypertensives, antiinfectives, antithrombotics, anxiolytics, and immunosuppressants.

In aggregate, available data demonstrate that some drugs exhibit a degree of in vivo removal by CS that may be clinically significant, whereas most show no, or only minimal removal (**Table 1**). In vivo evidence is available for a substantial portion of the investigated drugs. Antibiotics, cardiovascular drugs, and certain psychotropic agents are among the best-studied categories, whereas information for anaesthetics and antivirals remains limited. Expectedly there is substantial variability across different drugs underscoring the importance of individualized drug-specific assessments and further systematic evaluation in clinical settings.

**TABLE 1.**
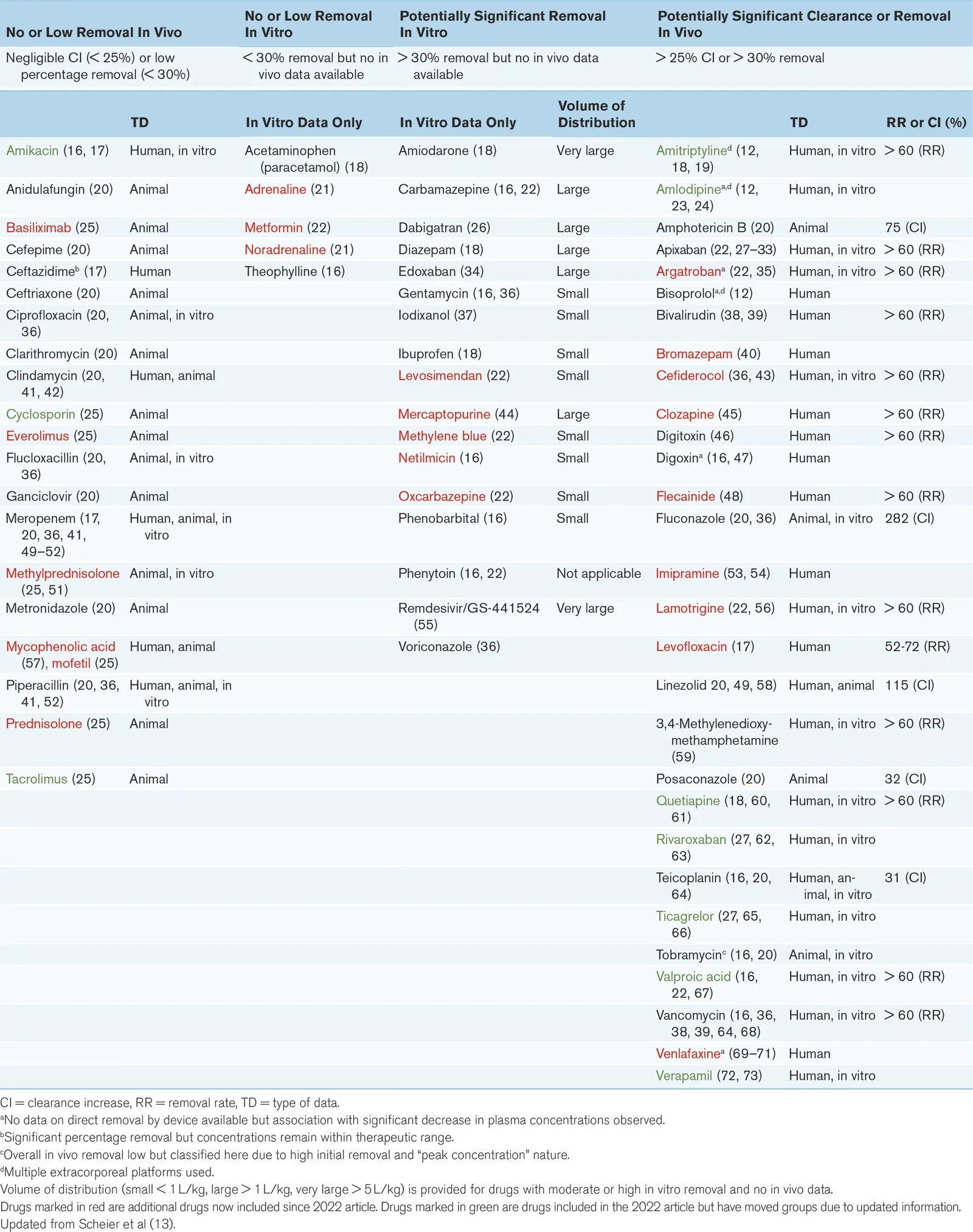
Classification of Drugs According to Clinical Significance of CytoSorb Adsorption

## DISCUSSION

### Adsorption Characteristics of the Device

The removal of substances by CS is primarily based on nonspecific hydrophobic interactions and substance concentration. Notably, adsorption occurs in a concentration-dependent manner. The greatest decreases in systemic drug concentration occur when agents are administered shortly before or during CS treatment initiation (13, 20, 74, 75). In general, a decline in CS clearance is observed over time likely related to the ongoing lowering of drug concentrations resulting in decreasing concentration gradients. Consequently, the timing of drug administration relative to active device use is critical to determine the degree of removal. Comparative data with other sorbent devices such as Jafron H.A. series suggest that, although the adsorption mechanism is in principle broadly similar, CS exhibits a more homogeneous pore distribution and consistent adsorption kinetics across a wide range of molecular weights (75). However, clinical evidence for these alternative systems remains limited, and most published PK data currently refer only to CS.

### Factors Influencing Drug Removal

**Figure 1.**
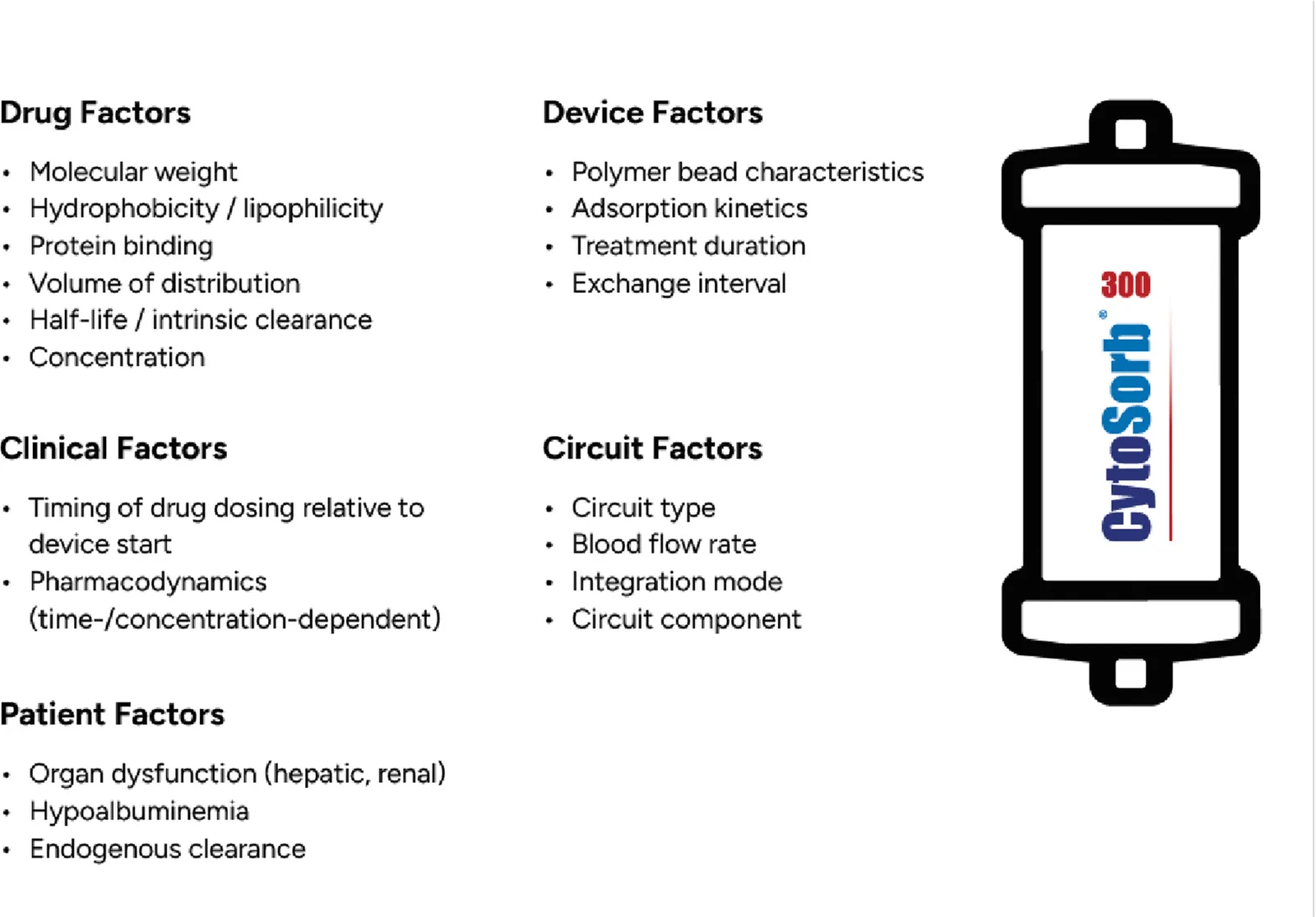
Factors influencing drug removal during CytoSorb therapy. Courtesy of CytoSorbents Europe GmbH.

PK data are usually derived from in vitro, animal or healthy volunteer studies and must therefore be interpreted with caution when extrapolated in the complex setting of critical illness. Of note, all CS in vivo studies were in critically ill patients (apart from cardiac surgery patients treated for antithrombotic removal only). Critically ill patients often exhibit profound physiologic derangements such as altered organ perfusion/function, fluid overload, hypoalbuminemia, acid-base disturbances, and systemic inflammation that may modify key PK parameters like volume of distribution (V_D_), protein binding, and intrinsic drug clearance (10). Therefore, multiple interdependent factors ranging from drug-specific characteristics to patient physiology and extracorporeal platform variables must be considered when determining the clinical relevance of potential CS-mediated drug removal (**Fig. 1**) (25).

Drugs with low V_D_ (< 1 L/kg) remain largely intravascular and therefore may be more susceptible to significant removal compared with drugs with larger V_D_ that distribute into peripheral tissues. Protein binding adds further complexity. Although a high protein binding limits the fraction of the free (adsorbable) entity of the drug, highly protein-bound drugs may still be subject to clinically relevant removal, depending on the dynamic equilibrium between free and bound drug fractions and the frequently observed reductions in albumin concentration in critically ill patients.

Blood flow may also significantly influence adsorption efficiency (68). When CS is integrated into CPB circuits, blood flow rates typically range around 500 mL/min (although not always monitored) (76), allowing for rapid equilibration and efficient removal (27, 77). In contrast, when integrated into CRRT, the device is exposed to substantially lower blood flow rates (100–250 mL/min) (78), resulting in a decreased but prolonged clearance process.

For drugs with high intrinsic clearance and short half-lives, even marked extracorporeal removal may not increase drug clearance significantly. Conversely, drugs with low endogenous clearance or prolonged half-life may undergo clinically relevant removal by CS even with only modest additional clearance. The above factors are the key reasons why caution should be exercised when extrapolating in vitro drug removal experiments to clinical settings since they are derived in controlled and reproducible conditions that do not replicate the complexity of in vivo physiology. Accordingly, in vitro data are particularly relevant when demonstrating lack of removal that establishes the inability of the polymer beads to adsorb the specific drug, and as such can be safely translated to the in vivo setting. Although we adopted a conservative “worst-case” approach by considering in vitro removal as potentially clinically relevant when in vivo data were lacking, it is important to emphasize that in vitro adsorption does not necessarily translate into meaningful in vivo loss of drug exposure (79). Therefore, the presence of in vitro adsorption should be interpreted primarily as a signal for potential removal rather than as proof of clinically relevant underexposure. In any case, drug removal by other extracorporeal circuits being active at the same time must also be considered independently from CS (16, 25, 80).

Beyond PKs, PD effects should also be considered. For concentration dependent drugs, such as aminoglycosides, anticonvulsants, or antidepressants, even modest extracorporeal clearance may impact efficacy. Conversely, for time-dependent antibiotics like β-lactams, reduced peak levels are often clinically irrelevant as long as concentrations remain above the minimum inhibitory concentration.

### Clinical Implications

An overview of the drugs most affected by CS therapy, distinguishing between intentional (i.e., antithrombotic removal surgery, or for removal for drug overdose) and unintentional drug removal (i.e., concomitant drugs administered during device use) is given in **Table 2**. The clinical relevance of any removal is highly context dependent, because the same drug elimination may be beneficial in one patient and undesirable in another. Of note, the present review aims to provide conservative guidance for dose adaptations, that is, to rather err on the side of caution in cases of drugs with incomplete or inconsistent data available.

**TABLE 2.** Drugs Cleared by CytoSorb With Potentially Significant In Vivo Removal (> 25% Clearance Increase or > 30% Removal) or Moderate-to-High In Vitro Removal (> 30%) but No In Vivo Data Available

Class (Intentional Removal in Case of Intoxication)	Substance	Class (Unintentional Removal)	Substance
Anticoagulants	Apixaban Dabigatran Edoxaban Rivaroxaban	Antiarrhythmics	Amiodarone Flecainide
Antidepressant (serotonin-norepinephrine-reuptake inhibitor)	Venlafaxine	Antibiotics	Cefiderocol Ceftazidime Gentamycin Levofloxacin Linezolid Netilmicin Teicoplanin Tobramycin Vancomycin
Antidepressants (tricyclic antidepressants)	Amitriptyline Imipramine		
Antiplatelet (P2Y12 inhibitor)	Ticagrelor		
Antipsychotics	Clozapine Quetiapine		
Cardiac glycoside	Digoxin Digitoxin		
Recreational drug	3,4-Methylenedioxymethamphetamine	Antidote/dye	Methylene blue
		Antiepileptics	Carbamazepine Lamotrigine Oxcarbazepine Phenobarbital Phenytoin Valproic acid
		Antifungal	Amphotericin B Fluconazole Posaconazole Voriconazole
		Antipsychotic	Bromazepam
		Antiviral	Remdesivir/GS-441524
		Benzodiazepine	Diazepam
		Beta-blocker	Bisoprolol
		Calcium channel blocker	Amlodipine Verapamil
		Contrast agent	Iodixanol
		Direct thrombin inhibitors	Argatroban Bivalirudin
		Inotrope	Levosimendan
		Nonsteroidal anti-inflammatory drug	Ibuprofen

### Unintentional Removal

**Figure 2.**
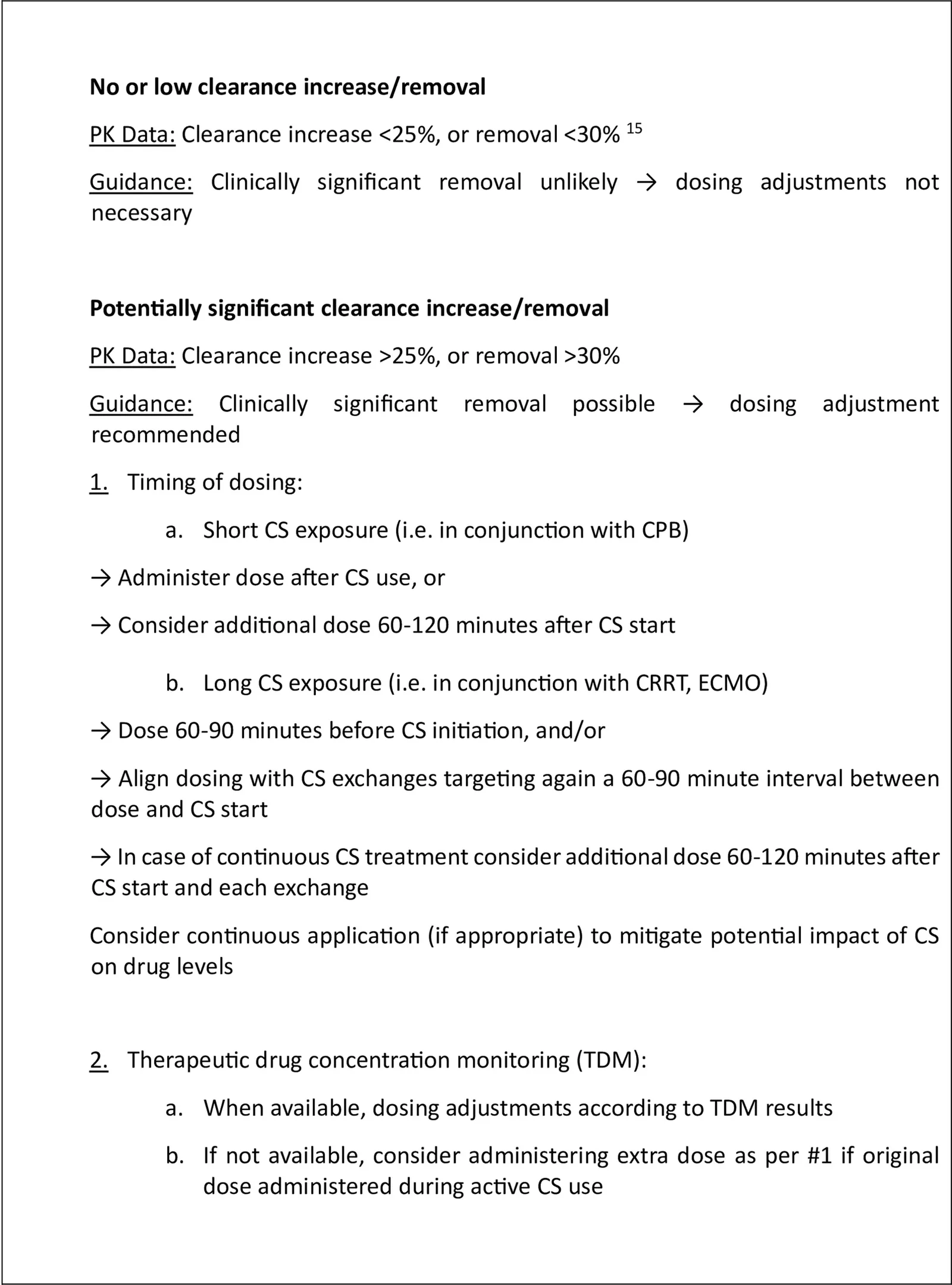
Guidance for minimization of unintended removal of concomitantly administered drugs during CytoSorb (CS) therapy. CPB = cardiopulmonary bypass, CRRT = continuous renal replacement therapy, ECMO = extracorporeal membrane oxygenation, PK = pharmacokinetic.

Based on available PK data and considering drug, patient, and extracorporeal circuit-specific aspects, general guidance for clinical practice has been developed as outlined in **Figure 2**. Although our framework ranks in vivo evidence above in vitro data, we intentionally did not differentiate the guidance by evidence type to avoid underestimation of extracorporeal drug removal. Because in vitro experiments do not capture endogenous clearance, physiologic protein binding and tissue distribution, they were considered conservatively as a worst-case estimate of maximum potential drug removal, recognizing that clinical impact could be lower in vivo. We further assume that drug concentrations can approach steady state within a 60–90 minutes CS-free interval, and we also take into account that any drug removal, if it occurs at all, is known to be most pronounced during the first 60–120 minutes after initiation (20). In cases of continuous CS treatment where interruptions for drug administration are not desired, additional dosing is recommended to mitigate the risk of subtherapeutic levels (58, 68).

In general, TDM remains strongly recommended when feasible and available to ensure safe and effective dosing, particularly in the case of drugs with narrow therapeutic windows or suspected susceptibility to removal (81). For drugs that are routinely titrated to clinical effect (i.e., analgesics, anaesthetics, vasopressors), the impact of any extracorporeal removal may be less critical. Dose management for these substances is not altered in the presence of CS therapy since administration remains guided by clinical effect. For other drugs dose adjustments can be guided by PD testing as is the case for antithrombotics using anticoagulation or platelet function testing.

Longer treatment durations or serial CS use as frequently encountered in critically ill ICU patients, can lead to cumulative clearance. In such settings supplemental doses after cartridge replacement may help maintain adequate serum and tissue levels of a drug. For concentration-dependent agents like aminoglycosides, early peak concentrations are critical and administration approximately 90 minutes before CS initiation or during device exchanges is an effective strategy to ensure sufficiently high peak levels. Subsequent potential removal by CS might even be beneficial to mitigate harmful drug side effects, for example, nephrotoxicity of aminoglycosides (20). Time-dependent drugs such as β-lactams require sustained plasma concentrations above the minimum inhibitory concentration for bacterial killing. In these cases adsorption may reduce peak concentrations but therapeutic concentrations are often maintained. For such agents, supplemental dosing or continuous application may be considered as a strategy to ensure efficacy. Importantly, the situation is not as complicated in cases of short device exposure (i.e., use during CPB) and can be addressed by administering drugs before the start or after the end of CPB.

### Intentional Removal

CS may be considered as an off-label adjunctive extracorporeal clearance strategy in select cases of severe drug intoxication or overdose, particularly when toxic or supra-therapeutic drug concentrations are present, the drug is poorly removed by conventional hemodialysis, no specific antidote is available or sufficient, and the patient has severe or life-threatening clinical manifestations despite standard supportive care. Efficacy should be monitored by serial drug concentration measurements whenever available, supported by PD markers where applicable and the patient’s clinical course, including hemodynamic, neurologic, bleeding, or organ-function parameters depending on the drug and indication. Potential risks include unintentional removal of concomitant medications, interactions with other extracorporeal circuits, and procedural risks inherent to extracorporeal blood purification. In general, CS therapy may be discontinued once drug concentrations have decreased to subtoxic or clinically acceptable levels and the patient has stabilized. Beyond the direct effect of drug removal from the circulation, CS may also contribute to the resolution of secondary complications of drug overdoses such as hyperinflammation, peripheral vasodilation and vasoplegia, rhabdomyolysis, and liver dysfunction, further supporting its role as a therapeutic adjunct in critical toxicological emergencies (53, 82, 83).

### Update From Previous Drug Removal Overview

As a significant addition to the safety profile of CS from the 2022 publication, new information was found for 24 additional drugs from various groups including antibiotics and immunosuppressants (marked in red in Table 1) (Scheier et al [13]). Ten previously included drugs now have additional in vivo information (previously in vitro), marked in green in Table 1, improving on the previous information.

- New drugs added: argatroban, basiliximab, bromazepam, cefiderocol, clozapine, epinephrine, everolimus, flecainide, imipramine, lamotrigine, levofloxacin, levosimendan, metformin, mercaptopurine, methylene blue, methylprednisolone, mofetil, mycophenolic acid, netilmicin, norepinephrine, oxcarbazepine, prednisolone, venlafaxine
- Drugs that now have additional in vivo data (both human and animal) and therefore moved category with updated information: amikacin, amitriptyline, amlodipine, cyclosporin, quetiapine, rivaroxaban, tacrolimus, ticagrelor, valproic acid, verapamil

### Gap Analysis

This review mapped the available evidence on CS-mediated drug removal and identified important gaps requiring further studies. Although data are now available on 72 drugs from 21 different classes, substantial gaps persist considering the vast drug armamentarium used in critical care and beyond. Despite the growing database, limited or no published data exist for several drugs related to the potential for removal by CS (**Table 3**); that said, for many of them this may not be a clinically relevant issue, as some drugs are titrated to effect, whereas others exceed the molecular weight cutoff of the device or lack hydrophobic properties and therefore are typically not prone to removal by CS. Future research should aim to quantify adsorption kinetics for a broader range of drugs under standardized clinical conditions. Development of predictive models integrating molecular size, protein binding, hydrophobicity, and extracorporeal flow dynamics could facilitate individualized dosing algorithms and optimize drug management during CS therapy.

**TABLE 3.** Drug Classes With Limited or No Published Data to Date

Antiarrhythmics	Sparse data beyond amiodarone and flecainide; other commonly used agents like lidocaine, procainamide, or sotalol are unstudied
Antibiotics	Sparse data on modern agents (cefiderocol included but others like ceftolozane-tazobactam, ceftaroline, or newer quinolones missing)
Antidiabetics	Sparse data (metformin only); no data on insulin, sodium-glucose cotransporter 2 inhibitors, glucagon-like peptide-1 agonists
Antiepileptics	Data on lamotrigine and carbamazepine; no data on levetiracetam, lacosamide
Antifungals	Limited to fluconazole, voriconazole, posaconazole; no data on echinocandins (micafungin, caspofungin)
Antipsychotics	Limited data beyond quetiapine and clozapine; no data on haloperidol, olanzapine, risperidone
Antithrombotics	Data on direct oral anticoagulants (apixaban, rivaroxaban, dabigatran) and P2Y_12_ inhibitor ticagrelor, but limited on other agents or combinations
Antivirals	Only remdesivir and ganciclovir addressed; no data on oseltamivir, acyclovir
Beta-blockers	Only bisoprolol and propranolol-like agents studied; no data on metoprolol, carvedilol, atenolol
Calcium channel blockers	Amlodipine and verapamil studied; no data on nifedipine, diltiazem
Cardiovascular inotropes/vasopressors	Limited to adrenaline, noradrenaline; no data on dopamine, vasopressin
Chemotherapeutics	No published data on chemotherapeutic agents (platinum derivatives, taxanes, anthracyclines, etc)
Hormonal agents	Sparse data; corticosteroids only partly covered
Immunosuppressants	Cyclosporin, tacrolimus, everolimus included; sirolimus, belatacept missing
Monoclonal antibodies and biologicals	No systematic data available on monoclonal antibodies (e.g., anti-tumor necrosis factor, anti-IL6, anti-CD20, immune checkpoint inhibitors)
Opioids	No data on fentanyl, sufentanil, remifentanil, or morphine
Neuromuscular blockers	No data on rocuronium, vecuronium, cisatracurium
Sedatives	No data on propofol, midazolam, dexmedetomidine

### Limitations

This review has several limitations. The gap analysis shows that for many commonly used drugs in the critical care setting data are not yet available, thereby precluding comprehensive guidance. If there are instances of conflicting data, we prioritized in vivo data over in vitro sources, potentially introducing bias. Furthermore, uses in cases of intoxication constitute off-label CS use as of the date of the present literature search, and treatment decisions ultimately lie at the discretion of the treating physician. No formal information specialist, librarian, or dedicated systematic review methodologist was involved in refinement of the search strategy, which may have limited the comprehensiveness of the literature search. To maintain consistency and comparability with the previously published review by Scheier et al (13), we used a similar search strategy and terminology, which may also have limited identification of additional relevant studies. Furthermore, this review was conducted as part of the manufacturer’s efforts to improve clinical awareness and to mitigate risk related to potential drug removal during CS therapy and was developed in collaboration with external subject matter experts. However, because several authors are affiliated with the manufacturer and some literature sources were identified through an industry-maintained database, the possibility of bias should be considered when interpreting the findings. Finally, given the heterogeneity and the remaining gaps in the available data, TDM is recommended whenever available to guide individualized patient care.

## CONCLUSIONS

CS hemoadsorption therapy may alter the PKs of various drugs, with clinical relevance depending on drug-, patient-, indication-, circuit-specific, and device-related factors. The evidence base has grown and now covers a wide spectrum of drug classes. Although this updated systematic review provides improved guidance for clinical practice including suggestions for dose adjustments and the role of therapeutic drug monitoring, individual patient factors including hepatic and renal dysfunction, inflammatory state, protein binding, volume status, and concurrent extracorporeal support must remain central to treatment decisions. Future research should focus on standardized in vivo studies, especially in under-represented drug classes, to better define the clinical relevance of adsorption across different extracorporeal settings. Accordingly, the main value of this review lies not only in summarizing current evidence, but also in highlighting where robust data are still lacking. Until such evidence is available, therapy should be guided by a combination of available data, clinical judgement, and close monitoring.

## Supplementary Material


