# Drug Clearance and Dosing During CytoSorb Hemoadsorption: A Systematic Review: Erratum

**DOI:** 10.1097/CCE.0000000000001470

**Published:** 2026-09-16

**Authors:** Teresa Klaus, Patrick M. Honoré, Gabriella Bottari, Silvia De Rosa, Detlef Kindgen-Milles, Antoine Schneider, Nandor Marczin, Harriet Adamson, Joerg Scheier, Efthymios N. Deliargyris, Thomas D. Nolin

In the article published in Volume 8, Issue 7 of *Critical Care Explorations*, Tables 1 and 2 contained errors related to heading alignment and the incorrect display of values. These errors have now been corrected in both the online and PDF versions of the article.

## References

[1] Klaus T Honoré PM Bottari G.: Drug clearance and dosing during cytosorb hemoadsorption: A systematic review. Crit Care Explor 2026;8(7): e1444 10.1097/CCE.0000000000001444 PMC13372516 42453077

