# Supplementary figures and images for: Drug Clearance and Dosing During CytoSorb Hemoadsorption: A Systematic Review
Source: Crit Care Explor. 2026 Jul 14;8(7):e1444. doi: 10.1097/CCE.0000000000001444 (PMC13372516; doi:10.1097/CCE.0000000000001444)

Supplemental Figure 1. PRISMA Chart

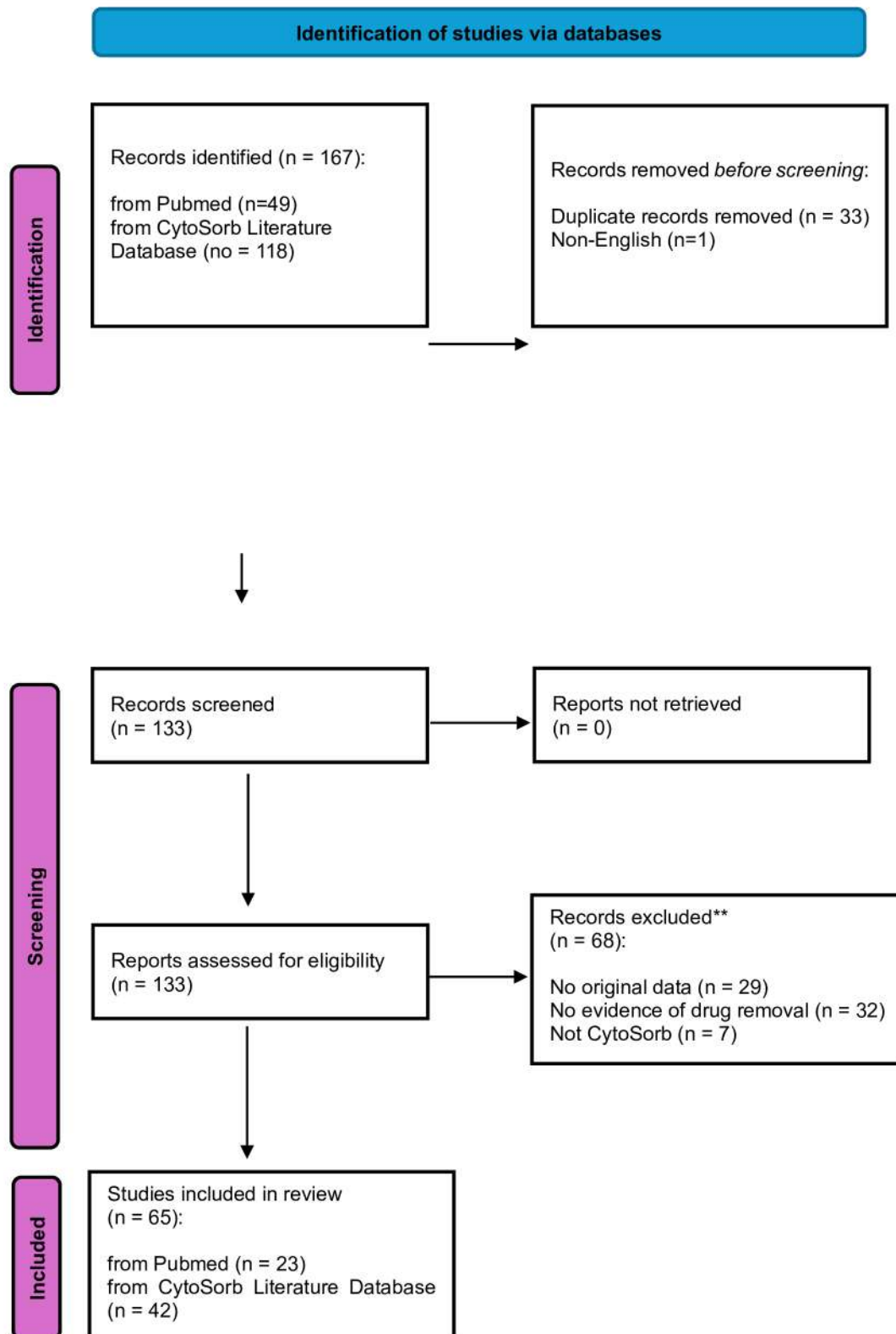

Supplement: Supplementary file 1 [file cc9-8-e1444-s001.pdf]
